# Supplementary material for: Brassinosteroid signaling-dependent root responses to prolonged elevated ambient temperature
Source: Nat Commun. 2017 Aug 21;8:309. doi: 10.1038/s41467-017-00355-4 (PMC5567177; doi:10.1038/s41467-017-00355-4)
Supplement: Supplementary file 1 — Supplementary Information_JWF ready [file 41467_2017_355_MOESM1_ESM.pdf]

File Name: Supplementary Information
Description: Supplementary Figures and Supplementary Table.

File Name: Supplementary Data 1
Description: Differential Expression of *Arabidopsis* root genes at 21°C and 26°C (FDR<0.05, induction/repression fold >1.3). RNA-seq was performed in triplicates on root RNA samples extracted from 10-day-old wild-type plants.

File Name: Supplementary Data 2
Description: List of defense-regulated genes and that are also regulated by elevated ambient temperature in roots.

File Name: Supplementary Data 3
Description: List of genes involved in metal homeostasis and that are also regulated by elevated ambient temperature in roots.

File Name: Supplementary Data 4
Description: List of genes regulated by both brassinosteroids and elevated ambient temperature in roots.

File Name: Peer Review File
Description:

# Supplementary information

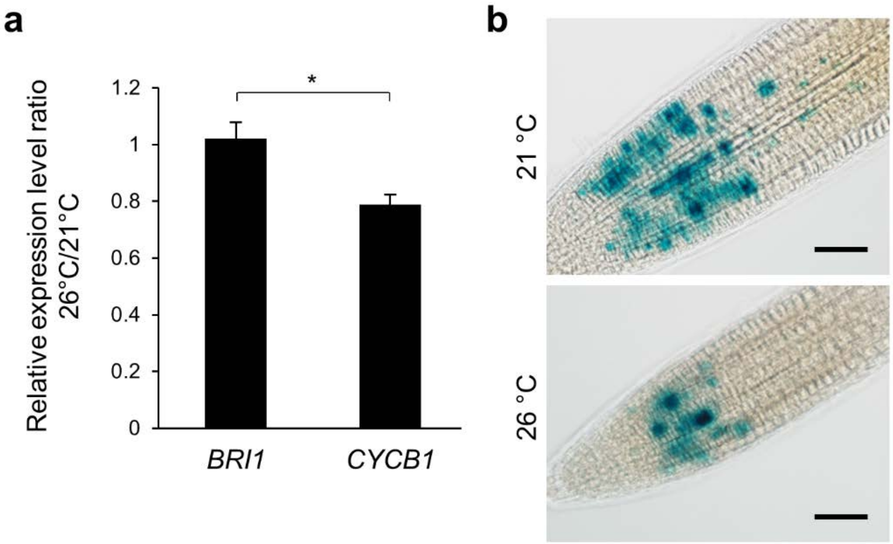


**Supplementary Figure 1. a,** Quantitative RT-PCR monitoring endogenous *BRI1* and *CYCB1;1* gene expression in roots from wild-type plants grown at 21°C or 26°C (mean+/-s.d., n=3). The asterisk indicates a statistically significant difference (t-test, $P<0.01$). **b,** Histochemical staining of the CYCB1;1::GUS cell cycle reporter line grown at 21°C or 26°C. Representative images are shown. Scale bar, 50 µm.

a

b

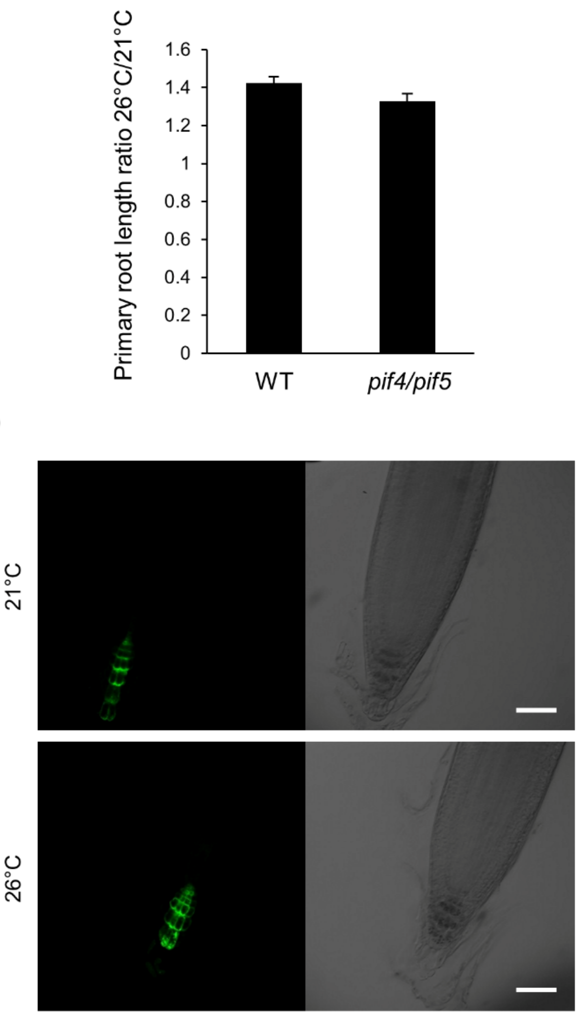


**Supplementary Figure 2. a,** Ratio of the primary root length from wild-type and *pif4/pif5* double mutant plants grown at 26°C over 21°C (mean+/-s.d., n=25). The difference in primary root length ratio between wild-type plants and *pif4/pif5* is not statistically significant. **b,** Influence of elevated growth temperature on auxin responses monitored by the DR5::GFP synthetic auxin reporter. Images correspond to Figure 2c, with a green LUT. Similar confocal detection settings were used to compare the two growth conditions. Scale bar, 50 µm.

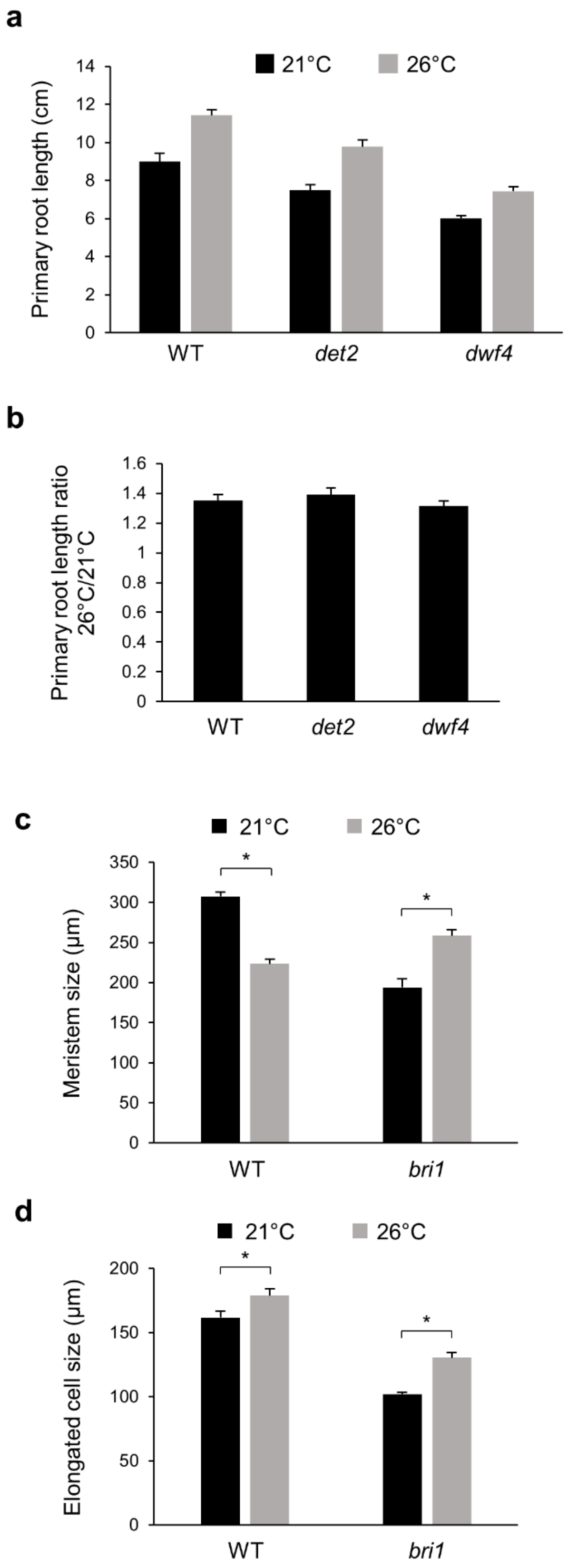


**Supplementary Figure 3. a,** Primary root length of 15-day-old wild-type, *det2* and *dwf4* mutant plants grown at 21°C or 26°C (mean+/-s.d., n=25). **b,** Ratio of the primary root length from wild-type, *det2* and *dwf4* grown at 26°C over 21°C (mean+/-s.d., n=25). The difference

in primary root length ratio between wild-type plants and both BR-deficient mutants is not statistically significant. **c,** Quantification of meristem size of wild-type and *bri1* mutant plants grown at 21°C or 26°C (mean+/-s.d., n=25). The asterisk indicates a statistically significant difference (t-test, $P<0.01$). **d,** Quantification of differentiated root epidermal cell length from wild-type and bri1 mutant plants grown at 21°C or 26°C (mean+/-s.d., n=25). The asterisk indicates a statistically significant difference (t-test, $P<0.01$).

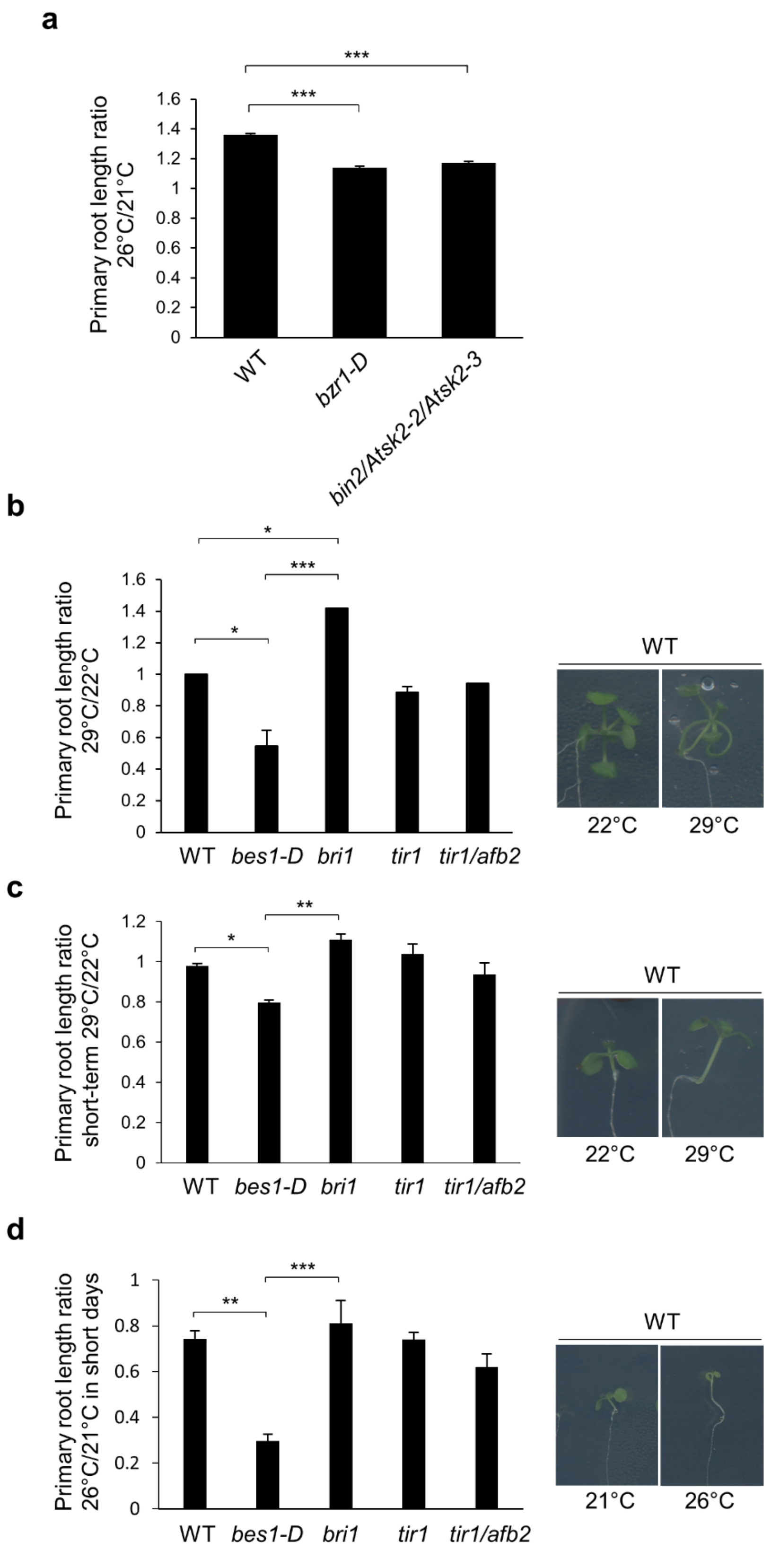


**Supplementary Figure 4. a,** Ratio of primary root length from 15-day-old wild-type (WT), *bzr1-D* and *bin2/Atsk2-2/Atsk2-3* mutants continuously grown 26°C/21°C (mean+/-s.d., n=3). The asterisks indicate a statistically significant difference (Kruskal-Wallis, P<0.0001). **b,** Ratio

of primary root length from 15-day-old wild-type (WT), *bes1-D*, *bri1*, *tir1* and *tir1/afb2* mutant plants continuously grown at 29°C/22°C (mean+/-s.d., n=3). The asterisks indicate a statistically significant difference (Kruskal-Wallis ; *, P<0.01 ; ***, P<0.0001). **c,** Ratio of primary root length from 9-day-old wild-type (WT), *bes1-D*, *bri1*, *tir1* and *tir1/afb2* mutant plants grown at 29°C/22°C (mean+/-s.d., n=3). Plants were cultivated for 5 days at 22°C and transferred for 4 additional days to 22°C or 29°C prior to scoring roots. The asterisks indicate a statistically significant difference (Kruskal-Wallis ; *, P<0.01 ; **, P<0.001). **d,** Ratio of primary root length from 15-day-old wild-type (WT), *bes1-D*, *bri1*, *tir1* and *tir1/afb2* continuously grown in short days at 29°C/22°C (mean+/-s.d., n=3). The asterisks indicate a statistically significant difference (Kruskal-Wallis ; **, P<0.001 ; ***, P<0.0001).

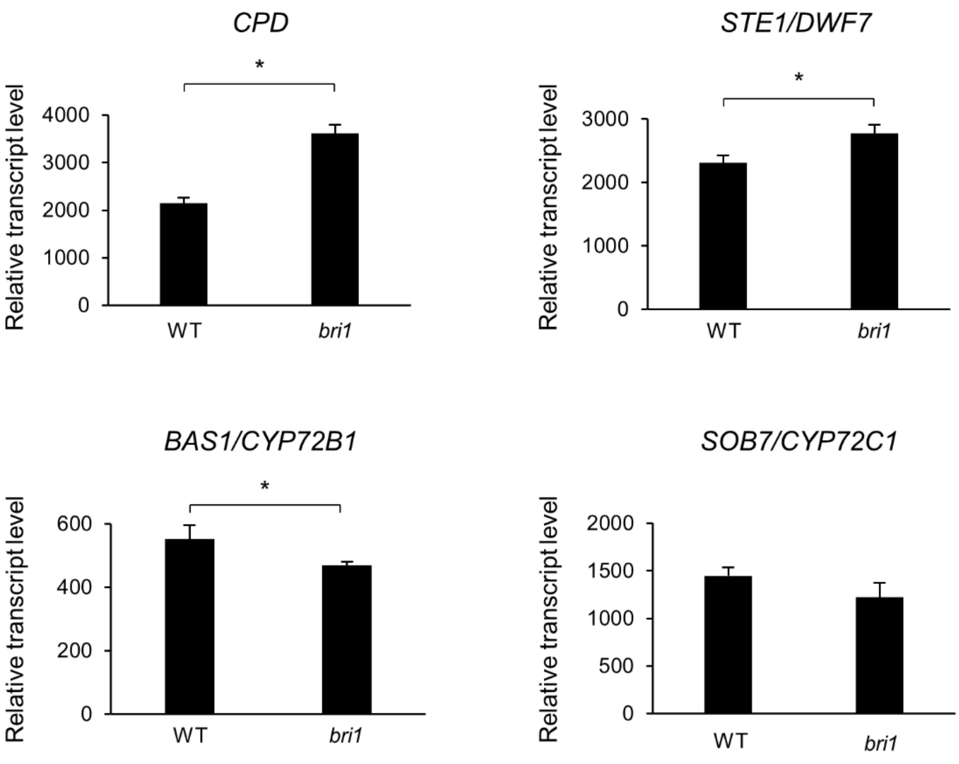


**Supplementary Figure 5.** Gene expression profiles of *CPD, STE1/DWF7, BAS1/CYP72B1 and SOB7/CYP72C1* in wild-type and *bri1* mutant (mean+/-s.d., n=3). The asterisks indicate a statistically significant difference (Mann-Whitney, P<0.01).

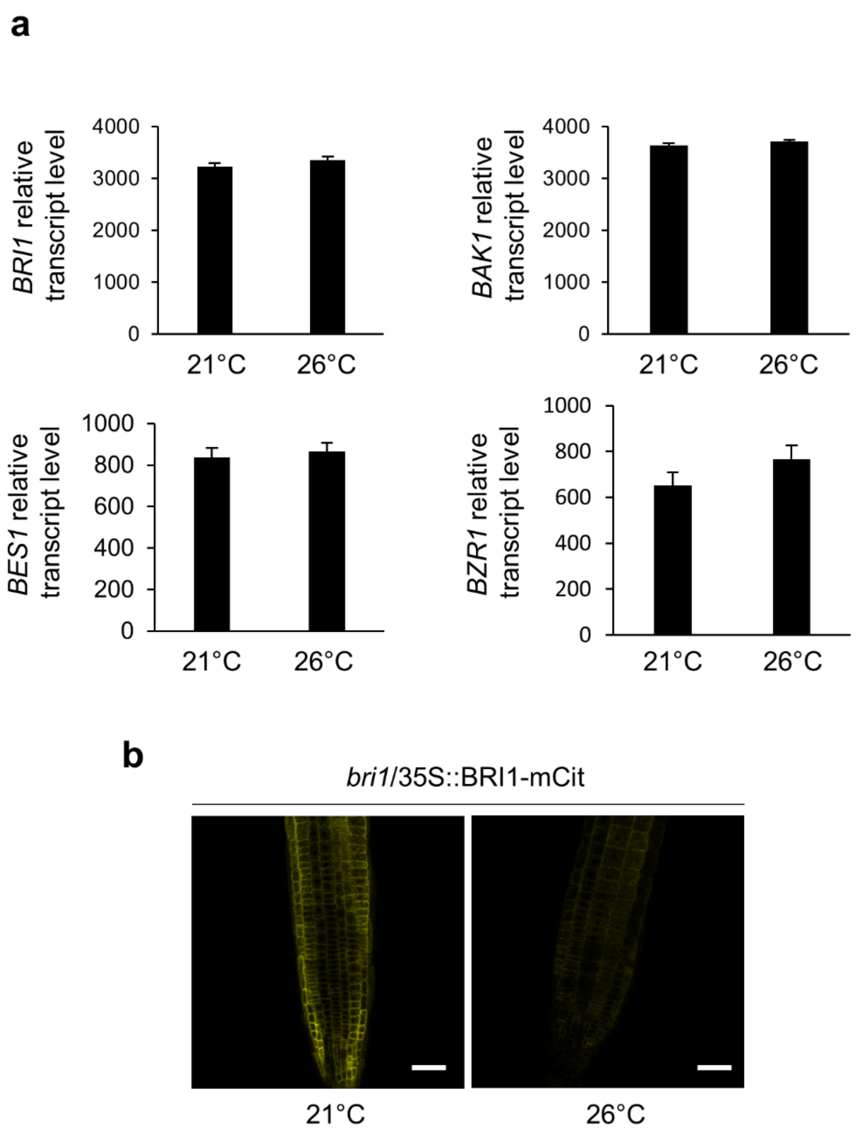


**Supplementary Figure 6. a,** Gene expression profiles of *BRI1*, *BAK1*, *BES1* and *BZR1* genes in roots from wild-type plants grown at 21°C or 26°C (mean+/-s.d., n=3). The data was extracted from the RNA-seq experiment. No statistical difference was observed between the two growth temperatures. **b,** Influence of increased growth temperature on constitutively-expressed BRI1-mCit. Similar confocal detection settings were used to compare the effect of temperature. Representative images are shown. Scale bar, 50 µm.

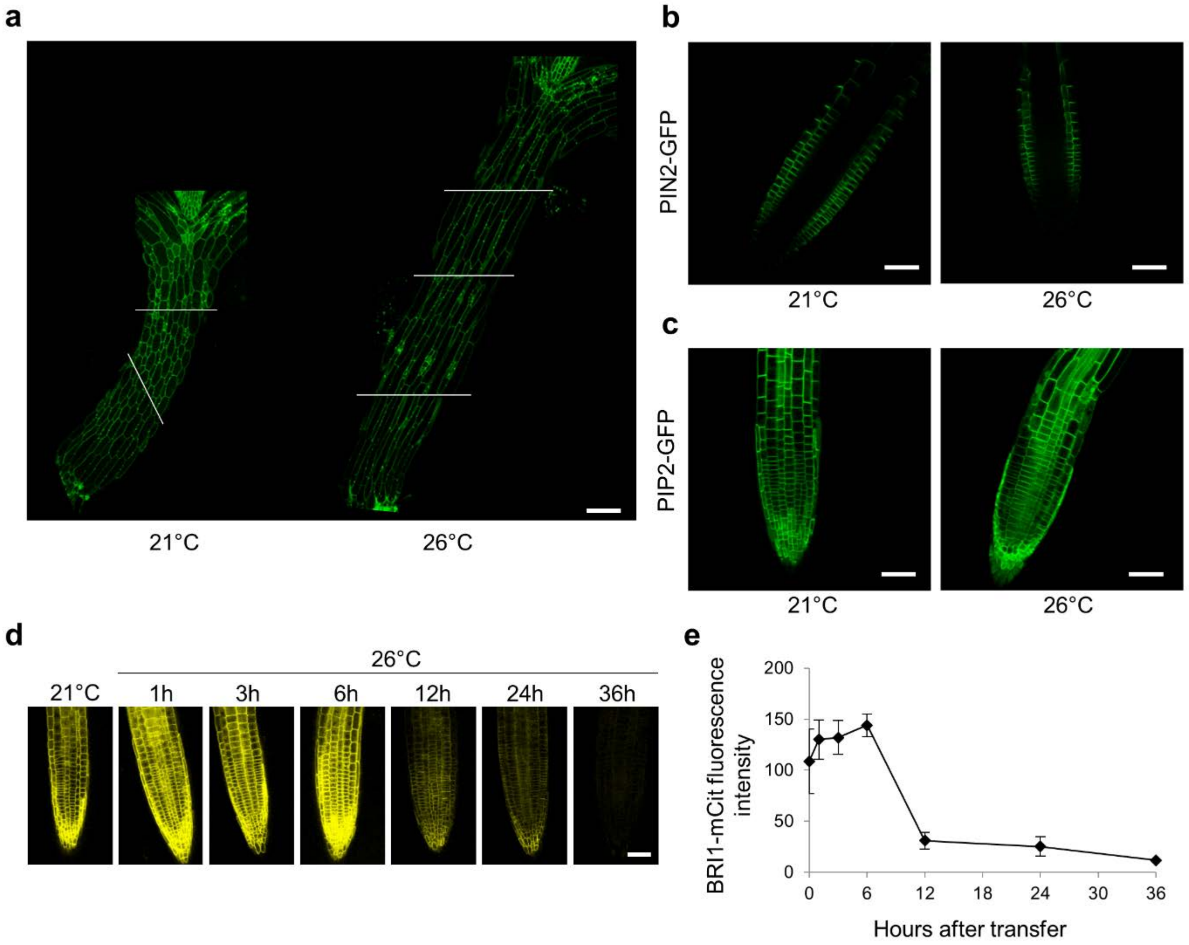


**Supplementary Figure 7. a,** Reconstitution of BRI1-mCitrine fluorescence in hypocotyls from plants grown at 21°C or 26°C. Similar detection settings were used to directly compare the effect of temperature on BRI1-mCit accumulation. Scale bar, 50 µm. **b, c,** Accumulation profile of PIN2-GFP (**b**) or PIP2;1-GFP (**c**) proteins in the primary root from plants grown at 21°C or 26°C. Similar detection settings were used between the two growth temperatures. Representative images are shown. Scale bar, 50 µm. **d,** Time-course analysis of BRI1-mCit levels in the root upon transfer from 21°C to 26°C. Similar detection settings were used during the course of this experiment. Representative images are shown. Scale bar, 50 µm. **e,** Quantification of BRI1-mCit levels upon transfer from 21°C to 26°C growth conditions (mean+/-s.d., n=6).

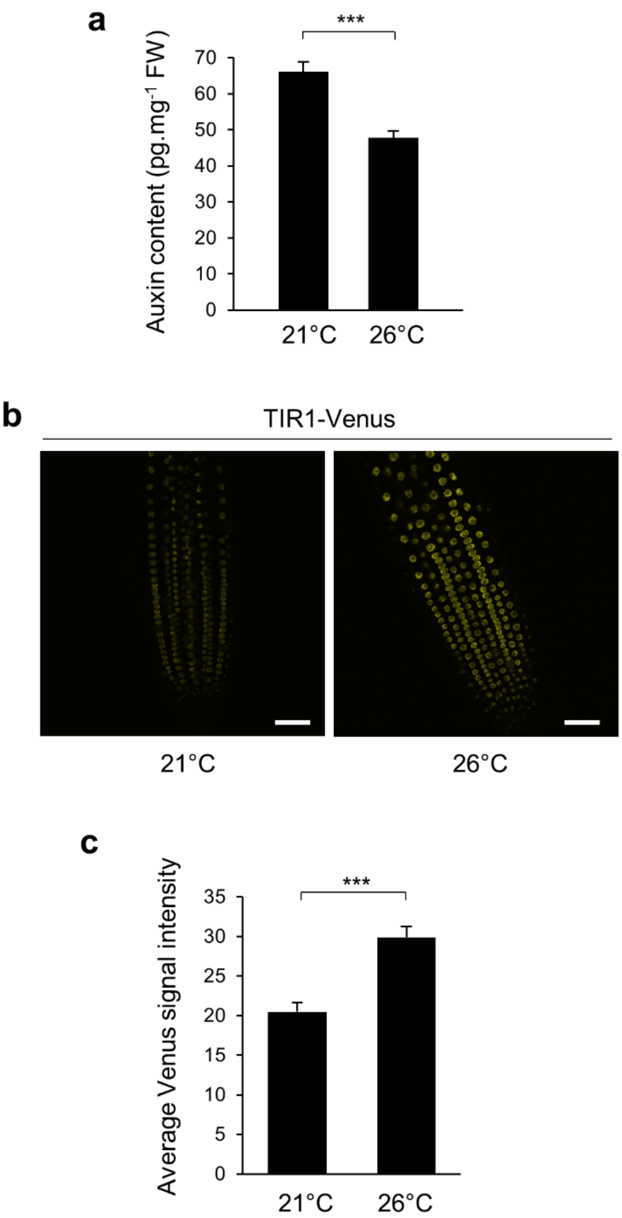


**Supplementary Figure 8. a,** Free auxin (IAA) measurements in 15-day-old roots from wild-type plants grown at 21°C or 26°C (mean+/-s.d., n=8). The asterisks indicate a statistically significant difference (Mann-Whitney, P<0.0001). **b,** Influence of continuous growth under elevated ambient temperature on TIR1-Venus protein accumulation in roots. Similar confocal detection settings were used to compare plants grown at 21°C and 26°C. Representative images are shown. Scale bar, 50 µm. **c,** Quantification of total root fluorescence from TIR1-Venus plants grown at 21°C and 26°C shown in (**b**) (mean+/-s.d., n=10). The asterisks indicate a statistically significant difference (Mann-Whitney, P<0.0001).

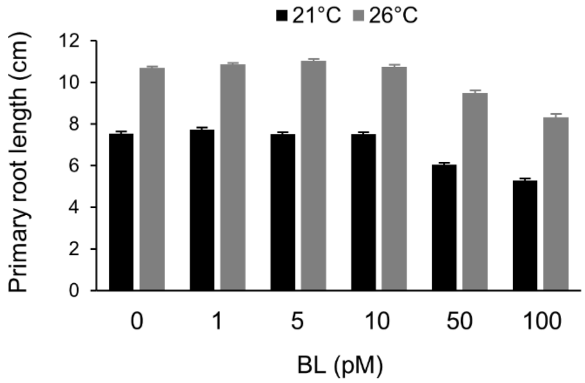


**Supplementary Figure 9.** Dose response analysis of primary root length to exogenously applied brassinolide (BL) in 15-day-old wild-type plants grown at 21°C or 26°C (mean+/-s.d., n=25).

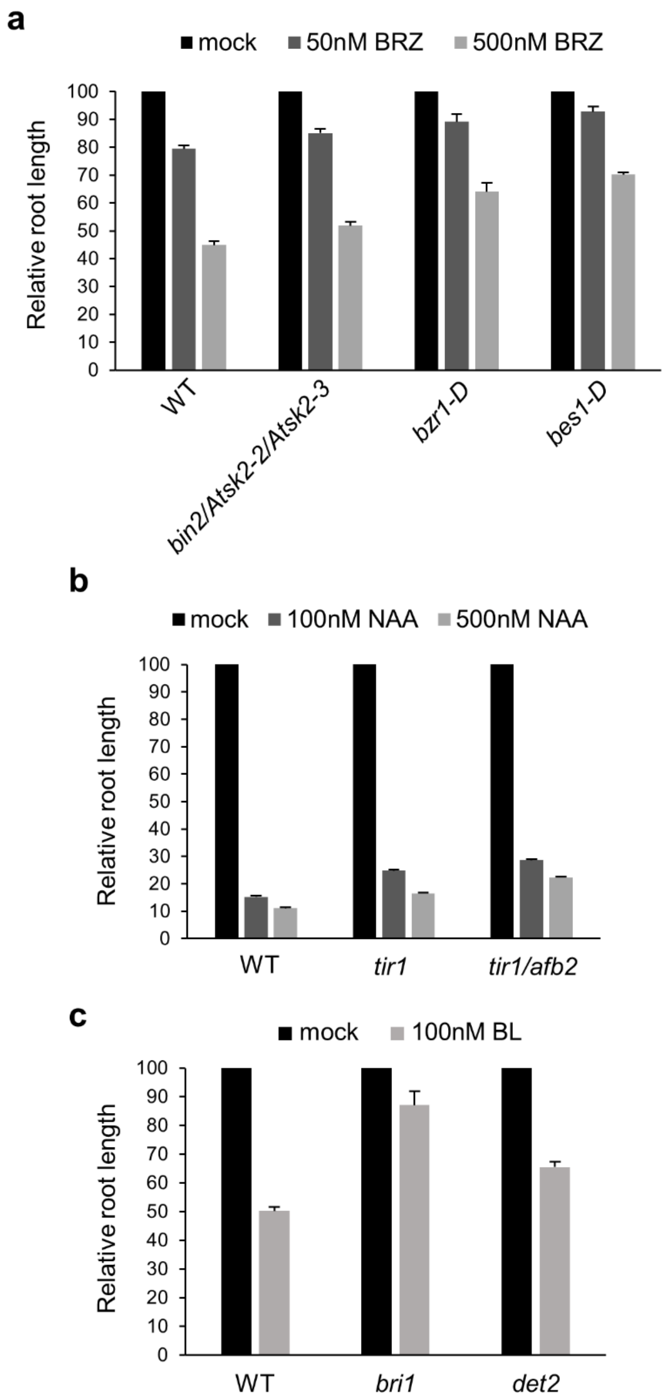


**Supplementary Figure 10. a,** Sensitivity of wild-type, *bin2/Atsk2-3/Atsk2-3*, *bzr1-D* and *bes1-D* to the BR biosynthetic inhibitor brassinazole (BRZ) (mean+/-s.d., n=25). Data are represented relative to mock for each genotype. **b,** Sensitivity of wild-type, *tir1* and *tir1/afb2* to auxin (NAA) (mean+/-s.d., n=25). Data are represented relative to mock for each genotype. **c,** Sensitivity of wild-type, *bri1* and *det2* to brassinolide (BL) (mean+/-s.d., n=25). Data are represented relative to mock for both genotypes.

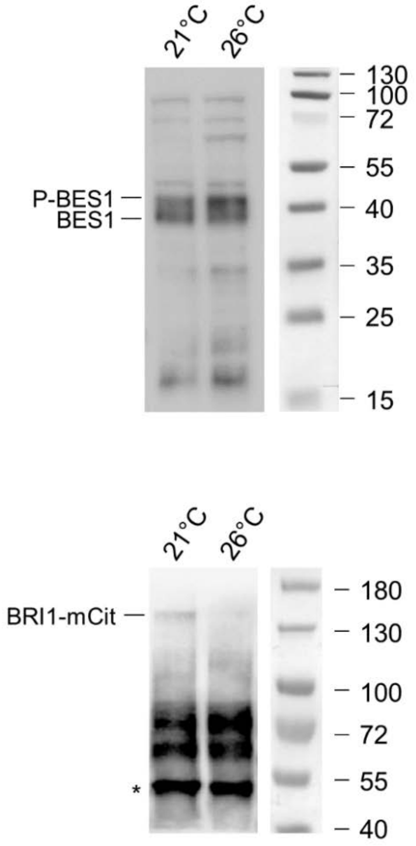


**Supplementary Figure 11.** Scans of full blots used in this study.

| **Primer name** | **Primer sequence** |
|---|---|
| CPD qPCR F | 5'-TTGCTCAACTCAAGGAAGAG-3' |
| CPD qPCR R | 5'-TGATGTTAGCCACTCGTAGC-3' |
| STE1 qPCR F | 5'-ACGTGGTTGGACCAAATGTT-3' |
| STE1 qPCR R | 5'-GATGGGTGGCATGGAGATAC-3' |
| BAS1 qPCR F | 5'-CCAAGGACCATGTCGTTAAGC-3' |
| BAS1 qPCR R | 5'-CCTGAAGTATAGCAAGATTCTGACC-3' |
| SOB7 qPCR F | 5'-GTCAGCAAAGAACTAAAGAATCC-3' |
| SOB7 qPCR R | 5'-GCTCGAATAGCAAGGAGACC-3' |
| CYCB1;1 qPCR F | 5'-TGCGTTTCTCTTGACTACAGGTTTT-3' |
| CYCB1;1 qPCR R | 5'-TCAATCATCGTCCTCGTACACG-3' |
| BRI1 qPCR F | 5'-AGCCGGGTCAGGGATAGATT-3' |
| BRI1 qPCR R | 5'-ACCCAAGGAAAATCGGACTGA-3' |
| PP2A qPCR F | 5'-AACGTGGCCAAAATGATGC-3' |
| PP2A qPCR R | 5'-GTTCTCCACAACCGCTTGGT-3' |

**Supplementary Table 1.** List of qPCR primers used in this study.
